# Telesurgery and the International Brazilian Journal of Urology in 2024

**DOI:** 10.1590/S1677-5538.IBJU.2024.06.01

**Published:** 2024-10-01

**Authors:** Luciano A. Favorito

**Affiliations:** 1 Universidade do Estado do Rio de Janeiro Unidade de Pesquisa Urogenital Rio de Janeiro RJ Brasil Unidade de Pesquisa Urogenital - Universidade do Estado do Rio de Janeiro - Uerj, Rio de Janeiro, RJ, Brasil; 2 Hospital Federal da Lagoa Rio de Janeiro RJ Brasil Serviço de Urologia, Hospital Federal da Lagoa, Rio de Janeiro, RJ, Brasil

The November-December number of Int Braz J Urol is the 31^nd^ under my supervision. In this number the Int Braz J Urol presents original contributions with a lot of interesting papers in different fields: Robotic Surgery, Prostate Cancer, Bladder Cancer, Kidney Cancer, Basic Research, Peyronie Disease, Endourology and Telesurgery. The papers came from many different countries such as Brazil, Italy, USA, Egypt and China, and as usual the editor's comment highlights some of them. The editor in chief would like to highlight the following works:

Dr. Amorim and collegues from Brazil, presented in page 670 (1) a nice systematic review about the retrograde intrarenal surgery with or without ureteral access sheath and concluded that ureteral access sheath (UAS) leads to a lower rate of post-operative fever and infection. However, UAS did not significantly reduce or increase the SFR or the rate of ureteral injuries during RIRS for patients with urolithiasis. The use of UAS should be considered to decrease the risk of infectious complications, particularly in those who may be at higher risk for such complications

Dr. Yang and collegues from China, performed in page 683 (2) a interesting systematic review about the robot-assisted radical cystectomy (RARC), laparoscopic radical cystectomy (LRC), and open radical cystectomy (ORC) in bladder cancer and concluded that LRC and RARC could be considered as a feasible and safe alternative to ORC for bladder cancer. Notably, compared with LRC, RARC may benefit from significantly lower transfusion rates, fewer complications and lower positive surgical margin rates. These data thus showed that RARC might improve the management of patients with muscle invasive or high-risk non-muscle invasive bladder cancer.

Dr. Mesquita and collegues from Brazil and USA performed in page 703 (3) a narrative review about the evidence of restorative therapies in the treatment of peyronie disease and concluded that restorative therapies has emerged as an innovative treatment option for PD and the results from current studies appear to be promising and demonstrated good safety profile. Unfortunately, due to scarce evidence, PRP and SCT are still considered experimental by American Urological Association (AUA) and European Association of Urology (EAU) guidelines. ESWT is recommended, by the same guidelines, for pain control only. More high-quality studies with long-term follow-up outcomes are needed to evaluate efficacy and reproducibility of those therapies.

Dr. Pellanda and collegues from Brazil, performed in page 714 (4) a interesting study about the endoscopic combined intrarenal surgery: best practices and future perspectives. Endourology is a very important topic with lot publications in Int Braz J Urol (5–8). In this study the authors concluded that Endoscopic Combined Intrarenal Surgery (ECIRS) demonstrates significant advantages in the management of large kidney stones. Future research should focus on well-designed randomized control trials to provide robust evidence of its efficacy, safety, and cost-effectiveness, potentially establishing ECIRS as the first option treatment for complex kidney stones.

Dr. Zhang and collegues from China, performed in page 727 (9) a nice study about Robotic-assisted radical nephroureterectomy using the KangDuo Surgical Robot-01 System versus the da Vinci System: a multicenter prospective randomized controlled trial and concluded that the KangDuo (KD)- Surgical Robot-01 (KD-SR-01) system is safe and effective for robot-assisted radical nephroureterectomy (RARNU) compared to the DV Si or Xi system. Further randomized controlled studies with larger sample sizes and longer durations are required. This paper is the cover of the present edition.

Dr. Kolanukuduru and collegues from Egypt performed in pag 737 (10) a very interesting study about the safety and efficacy of vacuum- assisted percutaneous nephrolithotomy (VmPCL) for the treatment of renal stone disease: an analysis of stone free status (SFR) and postoperative infections complications and concluded that vmPCNL is safe and efficacious, with an SFR of 74% at three months. The incidence of postoperative fever and SIRS/Sepsis is 5.5% and 2.9% respec- tively. Further randomized studies with large sample sizes are required to ascertain the rates of these complications in comparison to conventional approaches.

Dr. Moschovas and collegues from USA, permormed in page 754 (11) a very imporatant study about Telesurgery robotic-assisted radical prostatectomy using the Edge medical – a hot topic in urology. The authors concluded that as technological progress introduced novel robotic platforms and high-speed networks, the concept of Telesurgery became a tangible reality while 5G technology solved latency and transmission concerns. However, with these advancements, ethical consider- ations and regulatory frameworks should underline the importance of transparency and patient safety with responsible innovation in the field.

The Editor-in-chief expects everyone to enjoy reading.
